# Long-Acting Lipid-Lowering Injectables and Behavioral Moral Hazard: A Systematic Review of Adherence, Lifestyle Measurement, and Structural Evidence Gaps

**DOI:** 10.3390/healthcare14101319

**Published:** 2026-05-12

**Authors:** Carmen Marinela Cumpăt, Muthana Zouri, Nicoleta Zouri, Robert Daniel Negru, Andra Oancea, Bogdan Ionel Tamba

**Affiliations:** 1 Department of Medical Specialties III, Grigore T. Popa University of Medicine and Pharmacy, 700115 Iasi, Romania; marinela.cumpat@umfiasi.ro; 2 Department of Computer Science, Toronto Metropolitan University, Toronto, ON M5B 2K3, Canada; mzouri@torontomu.ca; 3 Faculty of ASENT3, Centennial College, Toronto, ON M1G 3T8, Canada; nzouri@centennialcollege.ca; 4 Department of Internal Medicine, Grigore T. Popa University of Medicine and Pharmacy, 700115 Iasi, Romania; robert.negru@umfiasi.ro; 5 Department of Medical Specialties I, Grigore T. Popa University of Medicine and Pharmacy, 700115 Iasi, Romania; 6 Advanced Centre for Research-Development in Experimental Medicine, Grigore T. Popa University of Medicine and Pharmacy, 700115 Iasi, Romania; bogdan.tamba@umfiasi.ro

**Keywords:** behavioral moral hazard, risk compensation, cardiovascular prevention, lipid-lowering therapy, treatment salience

## Abstract

**Background/Objectives:** Long-acting lipid-lowering injectable therapies, including PCSK9 monoclonal antibodies, inclisiran, and selected glucagon-like peptide-1 receptor agonists, represent a structural innovation in chronic disease management. By reducing dosing frequency and embedding pharmacologic persistence within healthcare delivery systems, these therapies address persistent challenges of long-term statin non-adherence. However, from a health economics perspective, such innovations may also alter incentive structures related to preventive behaviors. This study examines whether outcome measurement in the long-acting injectable literature reflects shifts in the balance between pharmacologic adherence and lifestyle-related prevention. **Methods:** A systematic review was conducted in accordance with PRISMA 2020 guidelines. Eligible studies included adult populations receiving PCSK9 monoclonal antibodies, inclisiran, or GLP-1 receptor agonists that reported pharmacologic adherence or persistence and/or lifestyle-related measures. Studies were categorized by therapy class and design. The prevalence of adherence and lifestyle outcome measurement was calculated, and a descriptive Measurement Asymmetry Index was defined as the difference between adherence measurement prevalence and lifestyle measurement prevalence. **Results:** Four studies met the inclusion criteria, comprising randomized controlled trials. Pharmacologic adherence or persistence was measured in all included studies (100%), whereas lifestyle-related outcomes were assessed in only one study (25%), limited to a GLP-1 receptor agonist trial. No study explicitly evaluated behavioral substitution, risk compensation, or changes in patient responsibility. The Measurement Asymmetry Index was 75 percentage points, indicating a pronounced imbalance between pharmacologic and behavioral outcome domains. **Conclusions:** The findings do not provide evidence of behavioral moral hazard at the individual level but reveal a structural asymmetry in how prevention is assessed within the injectable therapy evidence base. This pattern may reflect an emphasis on drug-attributable and measurable outcomes, with comparatively limited attention to lifestyle engagement. As long-acting therapies become more integrated into chronic disease management, incorporating standardized lifestyle metrics into cardiovascular research may be necessary to support a more balanced framework of preventive responsibility.

## 1. Introduction

Cardiovascular prevention has undergone a structural transformation over the past decade. Advances in lipid-lowering therapy have shifted management from daily oral pharmacotherapy toward long-acting injectable agents that substantially reduce treatment frequency and embed persistence within healthcare delivery systems. Proprotein convertase subtilisin/kexin type 9 (PCSK9) monoclonal antibodies demonstrated significant reductions in low-density lipoprotein cholesterol (LDL-C) and cardiovascular events in large randomized outcome trials, including FOURIER and ODYSSEY OUTCOMES [1,2]. These agents, administered biweekly or monthly, represented a departure from the behavioral demands of daily statin therapy.

Inclisiran further extends this trajectory through small interfering RNA (siRNA) technology that enables twice-yearly dosing following initial loading injections [3,4]. By dramatically reducing dosing frequency, inclisiran alters not only pharmacologic exposure but also the behavioral architecture of treatment adherence. Parallel developments in glucagon-like peptide-1 receptor agonists (GLP-1 RAs), such as once-weekly semaglutide [5], reflect a broader movement toward long-acting, biologically targeted therapies in cardiometabolic disease.

Although glucagon-like peptide-1 receptor agonists (GLP-1 RAs) are not primarily lipid-lowering agents, they are included in this review because they share key structural characteristics with PCSK9-targeted therapies and inclisiran, namely long-acting injectable delivery, reduced dosing frequency, and application in cardiometabolic populations with elevated cardiovascular risk. For example, once-weekly semaglutide has demonstrated efficacy in high-risk populations while similarly reducing the need for continuous patient-driven adherence [5]. From a behavioral economics perspective, these therapies collectively reduce the frequency of active patient engagement required for treatment persistence, thereby reshaping the incentive structure surrounding adherence and preventive effort [6,7]. As such, GLP-1 receptor agonists provide a relevant comparative context for examining how pharmacologic persistence and lifestyle engagement are measured across long-acting injectable therapies.

From a policy perspective, these innovations reflect intensified pharmacologic optimization of chronic disease management. Evaluations by the Institute for Clinical and Economic Review (ICER) underscore both the clinical effectiveness and substantial budgetary implications of these therapies [8]. Beyond clinical efficacy and cost, however, long-acting injectables reconfigure the locus of preventive effort: treatment persistence becomes less dependent on daily patient behavior and increasingly embedded within system-delivered care.

Long-term statin non-adherence has been widely recognized as a major barrier to effective cardiovascular prevention. Adherence to chronic medications frequently declines to approximately 50% within one year [6]. In cardiovascular populations, lower adherence is associated with significantly increased morbidity and mortality [7].

The economic consequences of non-adherence are substantial. Systematic reviews estimate that medication non-adherence generates significant avoidable healthcare expenditures due to preventable hospitalizations and disease progression [9]. Higher adherence has been associated with lower downstream healthcare utilization despite increased pharmaceutical spending [10,11].

In dyslipidemia, non-adherence reflects not only forgetfulness but the challenge of sustaining preventive effort when benefits are delayed and immediate costs, financial, behavioral, or cognitive, are present. Long-acting injectable therapies respond to this challenge by reducing the need for repeated daily action. In doing so, they restructure the incentive environment surrounding adherence. Persistence is less contingent on continuous patient engagement and more embedded within episodic clinical encounters.

The concept of moral hazard provides a useful analytical lens for examining such structural changes. In his foundational analysis, Pauly defined moral hazard as the predictable behavioral response that occurs when insurance coverage reduces the marginal cost of medical care [12]. Cutler and Zeckhauser later emphasized that moral hazard reflects changes in behavior after protection is obtained, with welfare implications depending on whether additional behavior is efficient or excessive [13].

While traditionally applied to utilization under insurance coverage, moral hazard can also be conceptualized behaviorally. When protection against adverse outcomes increases, through insurance, safety technologies, or biomedical innovation, individual incentives for preventive effort may shift. This form of behavioral moral hazard is closely related to the risk compensation literature, in which individuals adjust behavior in response to perceived reductions in risk.

In the context of lipid-lowering therapy, long-acting injectables may create a technologically mediated form of protection that reduces the salience of daily preventive action. This does not imply that such therapies cause behavioral substitution. Rather, it raises a structural question: when pharmacologic protection becomes more durable and less behaviorally demanding, how might incentives for complementary lifestyle engagement change?

Contemporary guidelines from the European Society of Cardiology (ESC/EAS) and the U.S. Preventive Services Task Force (USPSTF) continue to position lifestyle modification, diet, physical activity, and weight management as foundational components of dyslipidemia management alongside pharmacologic therapy [14,15]. These recommendations reflect a normative framework in which responsibility for cardiovascular risk reduction is shared between patients and healthcare systems.

Policy scholarship emphasizes that responsibility for health is distributed rather than purely individual [16,17]. Within value-based healthcare frameworks, efficient prevention depends on alignment between pharmacologic innovation and sustained behavioral engagement [18]. The emergence of long-acting injectable therapies raises the possibility that the locus of responsibility may be gradually shifting toward pharmacologic control.

This review focuses on patterns of outcome measurement rather than direct behavioral effects, using behavioral economic concepts as an interpretive framework.

Against this backdrop, the primary objective of this systematic review is to examine whether patterns of outcome measurement in studies of long-acting lipid-lowering therapies reflect such a shift. Specifically, we assess:

1. The extent to which pharmacologic adherence or persistence is measured in trials and real-world studies of PCSK9 monoclonal antibodies, inclisiran, and GLP-1 receptor agonists.
2. The extent to which lifestyle behaviors, diet, physical activity, or structured behavioral interventions are assessed.
3. Whether any studies explicitly evaluate behavioral substitution, risk compensation, or shifts in perceived patient responsibility.

The study does not assume the presence of behavioral moral hazard. Rather, it investigates whether a measurement asymmetry exists between pharmacologic persistence and lifestyle engagement. Such asymmetry does not only reflect differences in outcome prioritization; it also limits the capacity of the existing evidence base to detect behavioral moral hazard. In the absence of systematic lifestyle measurement, potential behavioral responses to long-acting pharmacologic protection remain empirically unobservable.

## 2. Theoretical Framework

### 2.1. Behavioral Moral Hazard

Behavioral moral hazard extends the traditional economic concept of moral hazard beyond financial utilization to changes in preventive effort. While classical moral hazard theory emphasizes increased utilization under reduced marginal cost [12,13], its logic also applies to preventive effort when perceived protection increases. While much of the literature focuses on increased service utilization, the concept can also be applied to shifts in individual preventive behavior when perceived protection increases.

Risk compensation theory provides a closely related framework. In prevention contexts, risk compensation has been debated in settings such as HIV pre-exposure prophylaxis (PrEP), where biomedical protection raised concerns about reduced precautionary behavior. Although empirical findings have been mixed, the theoretical mechanism, adjustment of behavior in response to perceived protection, remains analytically relevant. The key insight is not that protection necessarily reduces precaution, but that incentives for precaution may change when protection becomes more durable or externally provided.

In chronic disease management, preventive effort requires sustained behavioral engagement. Adherence to daily statin therapy, for example, depends on repeated patient action, and declines substantially over time [6,7]. Long-acting injectable therapies alter this structure by reducing the frequency with which patients must actively engage in medication-taking behavior. In doing so, they may influence the salience of cardiovascular risk and treatment in daily life.

Salience theory suggests that individuals allocate attention and effort to aspects of decision-making that are most cognitively prominent [19]. When preventive treatment is embedded in daily routines, it may serve as a recurring reminder of underlying health risk. Conversely, when pharmacologic protection is delivered intermittently and requires less ongoing effort, the perceived immediacy of risk may decline. Reduced salience may not eliminate preventive motivation, but it could modify the intensity or consistency of complementary lifestyle behaviors.

From a behavioral perspective, reduced dosing burden may alter the salience of preventive effort. When treatment becomes less behaviorally demanding and less embedded in daily routines, the visibility of cardiovascular risk management may decline. While this does not imply behavioral substitution, it suggests a potential pathway through which engagement with complementary lifestyle behaviors could be modified. In this review, this perspective is used as an interpretive lens rather than as a tested hypothesis.

### 2.2. Structural Moral Hazard and Outcome Measurement

Moral hazard may operate at two analytically distinct levels: individual behavior and institutional structure. In Pauly’s formulation, individuals adjust their consumption of medical care when insurance coverage reduces the marginal cost of treatment [12]. Cutler and Zeckhauser further clarified that moral hazard reflects incentive changes following protection, with welfare implications depending on whether resulting behavior is efficient or excessive [13]. In this traditional framework, moral hazard is analyzed at the level of the insured individual. In the context of long-acting lipid-lowering therapies, however, moral hazard may operate at two distinct levels: individual and structural.

Individual moral hazard refers to the possibility that patients, when protected by highly effective and long-acting pharmacologic therapies, may alter complementary preventive behaviors. Reduced dosing frequency could theoretically affect lifestyle engagement through changes in treatment salience, perceived risk, or effort allocation. This hypothesis remains empirical and is not presumed in this review.

Structural moral hazard, by contrast, concerns how healthcare systems, trial designs, and institutional incentives shape what is measured, incentivized, and defined as prevention. Long-acting injectable therapies are typically evaluated in large cardiovascular outcome trials, commonly supported by industry sponsors and designed to meet regulatory and reimbursement criteria. Adherence is routinely measured as a core indicator of treatment performance. Lifestyle behaviors, however, are rarely incorporated as trial endpoints outside obesity-focused GLP-1 studies.

System-level incentives may partially explain this pattern. Demonstrating pharmacologic efficacy, safety, and persistence is central to regulatory approval, reimbursement decisions, and health technology assessment. From a value-based care perspective, measurable outcomes that can be linked to pharmaceutical intervention are more readily quantified and attributed [13]. In contrast, lifestyle engagement is more difficult to standardize, measure, and attribute to specific therapeutic agents.

This creates a potential divergence between guideline expectations and trial measurement. ESC/EAS and USPSTF guidelines consistently position lifestyle modification as foundational in dyslipidemia management [14,15]. Prevention is conceptualized as a shared responsibility between patient behavior and pharmacologic therapy. Yet when clinical trials disproportionately measure pharmacologic persistence and omit structured assessment of lifestyle engagement, the empirical definition of prevention may narrow.

Structural moral hazard does not imply misconduct or inappropriate influence. Rather, it reflects how institutional incentives and research priorities can subtly redistribute responsibility within healthcare systems. If prevention becomes increasingly defined by pharmacologic continuation rather than sustained behavioral engagement, the locus of accountability may shift from patient effort to therapeutic design.

The present analysis does not assume that patients reduce lifestyle effort when treated with long-acting injectables. Instead, it evaluates whether patterns of outcome measurement signal a structural reallocation of responsibility within cardiovascular prevention.

## 3. Conceptual Model

**Figure 1:**
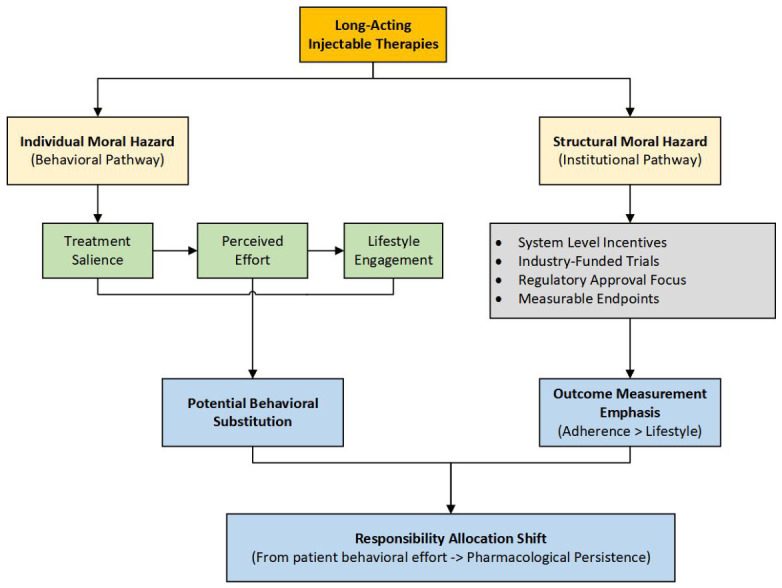
Conceptual Framework Linking Dosing Frequency, Measurement Prioritization, and Responsibility Allocation in Lipid-Lowering Therapy.

Figure 1 shows an integrated conceptual model linking dosing frequency, behavioural engagement, outcome measurement, and responsibility allocation in long-acting injectable therapies. The model illustrates two interacting pathways: an individual behavioural pathway, in which reduced treatment salience may influence engagement in lifestyle behaviours, and a structural pathway, in which system-level incentives shape outcome measurement priorities. The interaction of these pathways contributes to an observed emphasis on pharmacologic adherence relative to lifestyle engagement, with implications for the allocation of responsibility in cardiovascular prevention.

Lower treatment salience may influence behavioral engagement, including diet and physical activity, which remain foundational components of cardiovascular prevention. Changes in behavioral engagement may, in turn, shape responsibility allocation, reflecting the balance between patient-driven lifestyle management and pharmacologic control of risk.

Two contextual modifiers are incorporated into the model. Above the central pathway, Guideline Emphasis on Lifestyle (ESC, USPSTF) represents the normative expectation that lifestyle modification accompanies pharmacologic therapy. Below treatment salience, Trial Outcome Measurement Focus highlights the empirical prioritization observed in the current evidence base, where pharmacologic persistence is consistently measured.

To the right of the pathway, empirical findings from this review are integrated. Adherence measurement is frequently assessed and directly linked to responsibility allocation, whereas lifestyle measurement is rarely assessed. From a moral hazard perspective, this asymmetry does not establish behavioral substitution but suggests a potential structural shift in how responsibility for cardiovascular risk reduction is framed and monitored. The framework describes a conceptual interpretation of how reduced dosing burden and measurement prioritization may be related within the current evidence base.

## 4. Methods

This systematic review was conducted in accordance with the Preferred Reporting Items for Systematic Reviews and Meta-Analyses (PRISMA 2020) guidelines. A review protocol was developed a priori to define the research question, eligibility criteria, search strategy, and analytical approach; however, it was not registered in a publicly accessible database.

**Figure 2:**
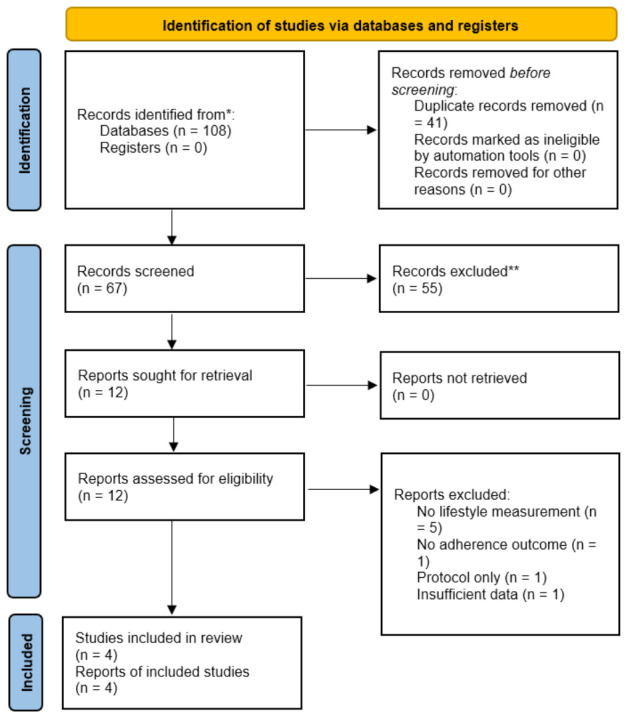
PRISMA 2020 flow diagram of study selection process. * Records identified from electronic database searches. ** Records were excluded after title and abstract screening because they did not meet the review eligibility criteria. The flow diagram was adapted from the PRISMA 2020 statement by Page et al. [20].

The protocol focused on long-acting lipid-lowering therapies and the assessment of pharmacologic adherence alongside lifestyle and behavioral outcomes. Any deviations from the protocol are described and justified within the Methods section. The study selection process is summarized in the PRISMA flow diagram (Figure 2).

Eligibility criteria were established in advance to align with the objective of examining adherence, lifestyle engagement, and potential behavioral moral hazard in the context of long-acting lipid-lowering therapies.

Studies were included if they involved adults (≥18 years) with hypercholesterolemia, established atherosclerotic cardiovascular disease (ASCVD), or elevated cardiovascular risk requiring lipid-lowering treatment. Studies focusing exclusively on pediatric populations, animal models, or non-cardiovascular conditions were excluded.

Eligible interventions included the following long-acting lipid-lowering therapies:

1. PCSK9 monoclonal antibodies including evolocumab (Biosynth, Louisville, KY, USA), and alirocumab (Biosynth, Louisville, KY, USA).
2. Inclisiran (Novartis Pharma AG, Basel, Switzerland).
3. GLP-1 receptor agonists when used in populations with cardiometabolic or cardiovascular risk such as semaglutide (Novo Nordisk, Bagsværd, Denmark).

Studies were eligible if they reported at least one of the following: pharmacologic adherence or persistence, including measures such as medication possession ratio, proportion of days covered, treatment continuation, injection completion, or discontinuation rates or lifestyle or behavioral measures, including dietary assessment, physical activity, structured lifestyle interventions, self-management behaviors, or discussion of behavioral engagement or substitution.

Studies that reported adherence outcomes without measuring lifestyle behaviors were retained, as identifying this measurement imbalance was central to the review.

Studies were excluded if they: focused solely on mechanistic, pharmacokinetic, or short-term LDL-C reduction without patient-level adherence or behavioral data, reported biochemical outcomes only, without adherence or behavioral measures, included exclusively pediatric or non-human populations, and lacked accessible full text.

A systematic search was conducted in PubMed, Embase, Web of Science, and Scopus from database inception to the final search date. The final search was conducted on 15 January 2026. The search strategy combined terms related to lipid-lowering therapies (including PCSK9 inhibitors, evolocumab, alirocumab, inclisiran, and GLP-1 receptor agonists), adherence and persistence (such as medication adherence, persistence, proportion of days covered, and discontinuation), and lifestyle or behavioral outcomes (including diet, physical activity, self-management, behavioral change, risk compensation, and moral hazard).

Controlled vocabulary terms were used where appropriate, and search strings were adapted for each database. The full electronic search strategies for each database, including all keywords and controlled vocabulary terms, are provided in Appendix A to ensure transparency and reproducibility. Reference lists of eligible articles, major clinical trials, and relevant guidelines were manually screened to identify additional studies.

The search was limited to English-language publications involving adult human populations. Duplicate records were removed prior to title and abstract screening. Study selection was performed independently by two reviewers. Titles and abstracts were screened for eligibility, followed by full-text assessment of potentially relevant studies. Discrepancies between reviewers were resolved through discussion and consensus.

Data were extracted using a standardized data extraction form developed for this review. Data extraction was conducted independently by two reviewers using the standardized form. Extracted variables were cross-checked for accuracy, and any discrepancies were resolved through discussion. The form captured study characteristics (authors, year, design, population, sample size, and follow-up duration), intervention details (drug class, dosing frequency, and administration mode), and outcome measures.

For each study, we extracted information on pharmacologic adherence or persistence, including measures such as medication possession ratio (MPR), proportion of days covered (PDC), continuation rates, injection completion, and discontinuation. We also recorded whether lifestyle or behavioral outcomes were assessed, including dietary behaviors, physical activity, structured lifestyle interventions, self-management measures, or discussion of behavioral engagement.

When lifestyle outcomes were not reported, this absence was explicitly coded, as identifying measurement asymmetry between pharmacologic adherence and lifestyle engagement was central to the review objective. Any explicit discussion of behavioral substitution, risk compensation, or patient responsibility was also documented.

The database search identified 108 records. After removal of 41 duplicates, 67 records were screened. Twelve full-text articles were assessed for eligibility, of which eight were excluded. Four studies met inclusion criteria.

### 4.1. Risk of Bias Assessment

The methodological quality of the included studies was assessed using the Cochrane Risk of Bias 2 (RoB 2) tool for randomized controlled trials. Each study was evaluated across the following domains: (1) bias arising from the randomization process, (2) bias due to deviations from intended interventions, (3) bias due to missing outcome data, (4) bias in measurement of outcomes, and (5) bias in selection of the reported result.

Risk of bias assessment was performed independently by two reviewers, with discrepancies resolved through discussion and consensus. The results of the assessment were summarized descriptively.

### 4.2. Analytical Approach

The analysis was designed to examine patterns of outcome measurement across long-acting lipid-lowering therapies and to assess potential asymmetry between pharmacologic adherence and lifestyle engagement. Given the heterogeneity of study designs and outcomes, a quantitative meta-analysis was not performed. Instead, a structured descriptive and comparative approach was used.

First, studies were classified by therapy type (PCSK9 monoclonal antibodies, PCSK9 siRNA, and GLP-1 receptor agonists). All included studies were randomized controlled trials. For each study, we identified whether pharmacologic adherence or persistence was measured, whether lifestyle behaviors (diet or physical activity) were assessed, and whether behavioral substitution or responsibility shift was explicitly evaluated.

Measurement prevalence was calculated as the proportion of studies reporting each outcome domain. A Measurement Asymmetry Index was defined as the difference between the prevalence of adherence measurement and the prevalence of lifestyle measurement within the included sample. This index was used descriptively as an exploratory indicator to illustrate differences in outcome prioritization across the included studies.

Comparisons were also made between therapy classes with respect to adherence and lifestyle outcome measurement. Findings were interpreted within a behavioral moral hazard framework, focusing on whether outcome reporting patterns may reflect differential emphasis on pharmacologic persistence versus lifestyle engagement. The analysis was descriptive and exploratory, and no causal inferences were drawn.

The analysis is descriptive and interpretive, and does not test behavioral hypotheses or causal relationships.

## 5. Results

### 5.1. Study Characteristics

The characteristics of the included studies are summarized in Table 1. All four included studies were randomized controlled trials (RCTs), including large, multicenter cardiovascular outcome trials evaluating PCSK9-targeted therapies and related agents. Although the review eligibility criteria permitted inclusion of observational and real-world studies, the final included evidence base consisted exclusively of randomized controlled trials. The RCTs were large, multicenter cardiovascular outcome trials evaluating PCSK9-targeted therapies.

Sample sizes varied substantially. Major outcome trials enrolled large populations exceeding 15,000 participants, whereas other studies included several thousand participants.

Follow-up duration ranged from approximately 12 months to more than 2 years across the included randomized controlled trials. Some inclisiran extension data reported follow-up extending to 4 years, although treatment adherence in those settings remained protocol-driven.

### 5.2. Risk of Bias

The included randomized controlled trials were judged to be at low to moderate risk of bias. Large cardiovascular outcome trials demonstrated low risk across most domains, particularly in relation to randomization and outcome measurement. Some concerns were noted regarding the potential for bias due to deviations from intended interventions and the reporting of adherence outcomes under protocol-driven conditions. However, no study was judged to be at high risk of bias (Table 2).

### 5.3. Adherence–Lifestyle Measurement Patterns

Pharmacologic adherence or persistence was measured in all four studies (100%). Adherence was assessed using study-specific metrics, including scheduled injection completion or treatment continuation. None of the studies reported standardized administrative adherence measures such as proportion of days covered (PDC) or medication possession ratio (MPR).

In contrast, lifestyle measurement was limited. Only one study (25%) assessed any lifestyle-related variable, including diet, physical activity, or structured behavioral intervention. No study explicitly evaluated behavioral substitution, risk compensation, or changes in patient self-management responsibility.

When stratified by therapy class, differences in measurement patterns were observed (Table 3). The single GLP-1 receptor agonist study incorporated dietary and physical activity assessment and included a structured behavioral program. In contrast, none of the studies evaluating PCSK9 monoclonal antibodies or inclisiran reported measurement of diet, physical activity, or structured behavioral engagement. Importantly, the absence of lifestyle measurement across most studies does not merely reflect omission but represents a systematic gap that constrains the ability of the literature to evaluate behavioral responses to long-acting therapies.

The difference between adherence measurement prevalence (100%) and lifestyle measurement prevalence (25%) yielded a Measurement Asymmetry Index of 75 percentage points within the sample, interpreted as a descriptive indicator of outcome measurement imbalance.

### 5.4. Guideline Comparison

Despite universal reporting of pharmacologic adherence, lifestyle assessment was uncommon. This contrasts with major cardiovascular prevention guidelines, including those from the European Society of Cardiology (ESC) and the U.S. Preventive Services Task Force (USPSTF), which emphasize lifestyle modification as a foundational component of lipid management alongside pharmacologic therapy.

Within the analyzed studies, lifestyle engagement was rarely measured, and no study evaluated behavioral substitution or shifts in responsibility. This divergence reflects a difference between guideline recommendations and outcome reporting patterns in injectable lipid-lowering therapy studies.

## 6. Discussion

This systematic review examined whether patterns of outcome measurement in studies of long-acting lipid-lowering injectable therapies reflect a redistribution of preventive responsibility. Across the included studies, pharmacologic adherence or persistence was consistently assessed, whereas lifestyle behaviors were rarely measured, and no study explicitly evaluated behavioral substitution or risk compensation. This imbalance suggests that pharmacologic persistence may serve as the primary operational indicator of preventive success within the current evidence base. As a result, an important epistemic limitation arises: without systematic assessment of lifestyle engagement, the literature cannot meaningfully evaluate the presence or absence of behavioral moral hazard. Given the limited number of included studies, these findings should be interpreted as exploratory and hypothesis-generating rather than definitive evidence of structural effects.

From the perspective of moral hazard theory, these findings may reflect a potential structural pattern, although this interpretation remains hypothesis-generating and requires further empirical validation. By reducing dosing burden, long-acting injectable therapies shift adherence from continuous patient-driven action to episodic clinical encounters, potentially diminishing the salience of preventive effort. This interpretation offers a theoretical lens through which the observed emphasis on pharmacologic outcomes can be understood.

### Measurement Asymmetry as an Interpretive Framework

Building on the behavioral moral hazard perspective, this review uses the concept of measurement asymmetry to interpret how structural changes in dosing burden relate to outcome reporting patterns. The inclusion of GLP-1 receptor agonists reflects a conceptual grouping based on treatment structure rather than pharmacologic class, emphasizing shared features such as long-acting injectable delivery and reduced adherence burden. This broader framing enables comparison of how outcome measurement and preventive responsibility are defined across structurally similar, though clinically distinct, therapeutic approaches. In direct relation to the study objectives, the findings demonstrate that pharmacologic adherence or persistence is consistently measured across trials of PCSK9 monoclonal antibodies, inclisiran, and related therapies, whereas lifestyle behaviors, including diet, physical activity, and structured behavioral interventions, are assessed infrequently. Moreover, no included studies explicitly evaluate behavioral substitution, risk compensation, or shifts in perceived patient responsibility.

Reductions in dosing burden, achieved through biweekly, monthly, or biannual injectable therapies, reshape the behavioral structure of treatment adherence. Daily statin use requires repeated patient action [6], whereas long-acting injectables embed persistence within episodic clinical encounters. As treatment becomes less integrated into everyday routines, the salience of cardiovascular risk management may diminish, with potential implications for engagement in complementary preventive behaviors. Treatment salience is not solely determined by dosing frequency but is shaped by clinical context, patient–provider interaction, and the symbolic meaning of therapy, which may either attenuate or reinforce engagement with preventive behaviors.

Within this context, pharmacologic adherence emerges as the dominant operational indicator of treatment success, while lifestyle engagement remains underrepresented in outcome measurement. Clinical guidelines consistently emphasize that pharmacologic therapy and lifestyle modification are complementary components of cardiovascular prevention [14,15]. However, when empirical evaluation prioritizes drug-attributable outcomes, the measurement of preventive effort becomes asymmetrically aligned with pharmacologic persistence. Such measurement asymmetry does not constitute evidence of behavioral substitution but reflects how preventive success is defined within the evidence base.

These findings are particularly relevant to the third objective of this review. The absence of explicit evaluation of behavioral substitution or risk compensation does not indicate that such effects are absent, but rather that they are not empirically observable within the current literature. If changes in lifestyle engagement were occurring, their detection would depend on systematic behavioral assessment. The lack of structured lifestyle measurement therefore limits the capacity of existing studies to assess potential shifts in patient behavior or perceived responsibility.

From a health systems perspective, this pattern may be understood within a framework of structural moral hazard. Regulatory approval and reimbursement decisions prioritize quantifiable, drug-attributable outcomes such as LDL-C reduction, cardiovascular events, and treatment persistence. In contrast, lifestyle behaviors are multidimensional, less standardized, and more difficult to attribute to specific interventions. Institutional incentives may therefore favor pharmacologic endpoints, narrowing the operational definition of prevention toward adherence-based metrics rather than sustained behavioral engagement.

At the same time, the clinical rationale for long-acting injectable therapies remains clear. Non-adherence to chronic cardiovascular medication is well-documented and associated with adverse outcomes [6,7]. Reducing dosing frequency may improve persistence and thereby enhance population-level cardiovascular risk reduction. In this sense, long-acting therapies address a genuine adherence challenge. The present findings should therefore be interpreted not as a critique of pharmacologic innovation, but as an indication that outcome measurement has not fully captured the broader scope of preventive care.

The alignment of findings across all three objectives highlights a structural imbalance in the evidence base: pharmacologic adherence is systematically measured, lifestyle engagement is inconsistently assessed, and behavioral responses are not directly evaluated. This imbalance suggests that the current literature is limited in its ability to fully characterize preventive behavior in the context of long-acting lipid-lowering therapies and underscores the importance of integrating behavioral metrics into future study designs.

## 7. Limitations

Several limitations should be considered when interpreting the findings of this review. First, the number of included studies was limited, reflecting the relatively recent introduction of long-acting lipid-lowering injectable therapies and the specificity of the eligibility criteria. The focus on studies that reported adherence or persistence outcomes constrained the pool of eligible trials. As a result, the sample size was modest and may not capture the full diversity of ongoing research in this rapidly evolving field. The small number of included studies limits the ability to draw definitive conclusions regarding structural moral hazard or shifts in preventive responsibility. The interpretations proposed in this review should therefore be viewed as hypothesis-generating and exploratory.

Second, heterogeneity in study design, populations, and outcome definitions limited the ability to conduct quantitative synthesis. Randomized cardiovascular outcome trials differed in follow-up duration, adherence assessment methods, and reporting practices. Adherence was typically measured using protocol-driven injection completion or treatment continuation rather than standardized metrics such as proportion of days covered (PDC) or medication possession ratio (MPR). This variability restricts cross-study comparability and limits the precision with which adherence patterns can be contrasted across therapeutic classes.

Third, the Measurement Asymmetry Index developed in this review is descriptive and exploratory rather than inferential. It reflects patterns within a small sample of studies and is intended as a heuristic indicator of outcome prioritization rather than a generalizable quantitative measure. The index captures the difference in reporting prevalence between pharmacologic adherence and lifestyle engagement outcomes. It does not establish that reduced dosing burden causes diminished lifestyle effort, nor does it demonstrate behavioral substitution at the patient level. Moral hazard theory, as articulated by Pauly and further refined by Cutler and Zeckhauser, describes incentive-based behavioral responses [12,13]. The present study examines outcome measurement architecture rather than observed patient behavior. Accordingly, conclusions pertain to structural patterns in reporting rather than to confirmed behavioral effects.

Fourth, absence of reported lifestyle measurement in trial publications does not necessarily imply absence of lifestyle counseling in clinical practice. Clinical guidelines from the ESC/EAS and USPSTF continue to emphasize lifestyle modification as foundational to dyslipidemia management [14,15]. Clinicians may provide behavioral counseling even if such engagement is not systematically captured in trial endpoints. Publication practices and reporting priorities may therefore underestimate the extent of behavioral reinforcement occurring in routine care.

Fifth, this review is limited to published literature and may be subject to reporting bias. Clinical trials are typically designed to meet regulatory and reimbursement requirements that prioritize measurable, drug-attributable outcomes. As a result, pharmacologic adherence and clinical endpoints are consistently reported, whereas lifestyle behaviors, being multifactorial and less directly attributable to specific interventions, may be underreported even when assessed. This structural tendency in outcome reporting complicates the interpretation of observed measurement asymmetry.

Finally, the conceptual perspectives used in this review are interpretive and not empirically tested within the included studies. Although informed by behavioral economic theory, including moral hazard and salience [12,13,19], they are used to contextualize patterns of outcome measurement rather than to establish causal relationships. Future research incorporating standardized lifestyle metrics and longitudinal designs would be necessary to evaluate potential relationships between dosing frequency and preventive engagement.

## 8. Conclusions

Long-acting lipid-lowering injectable therapies represent an important pharmacologic advance in cardiovascular prevention, addressing the well-documented challenge of long-term medication non-adherence [6,7]. By reducing dosing burden and embedding persistence within clinical delivery systems, these therapies offer a structural solution to daily adherence decline. However, their emergence also invites examination through the lens of moral hazard theory.

This review does not demonstrate behavioral moral hazard at the patient level. Rather, it identifies a consistent measurement asymmetry in the existing evidence base: pharmacologic adherence is universally assessed, while lifestyle engagement is infrequently measured and behavioral substitution is not explicitly evaluated. Interpreted within the framework articulated by Pauly and Cutler and Zeckhauser [12,13], this review does not establish structural moral hazard but identifies patterns in outcome measurement that may suggest a potential shift in how preventive responsibility is framed. These findings should be interpreted as exploratory and hypothesis-generating, highlighting the need for future research incorporating standardized behavioral measures.

Contemporary clinical guidelines emphasize lifestyle modification as foundational to dyslipidemia management [14,15]. Ensuring that behavioral engagement remains visible within trial design and outcome reporting is therefore critical to maintaining the shared framework of prevention endorsed by these frameworks. Integrating standardized lifestyle metrics into future cardiovascular outcome studies of long-acting therapies would strengthen alignment between pharmacologic innovation and preventive intent.

In extending moral hazard theory from financial utilization to the architecture of outcome measurement in chronic disease prevention, this review highlights the importance of examining not only what therapies do, but also how prevention is defined, measured, and implemented. As cardiovascular pharmacotherapy continues to evolve, preserving the balance between technological protection and sustained behavioral engagement will remain central to equitable and effective prevention policy.

## Figures and Tables

**Table 1:** Injectable Therapy Study Characteristics.

Variable	Ray_2020 [4]	Sabatine_2017 [1]	Schwartz_2018 [2]	Wilding_2021 [5]
Therapy_Class	PCSK9 siRNA	PCSK9 mAb	PCSK9 mAb	GLP-1 RA
Drug_Name	Inclisiran	Evolocumab	Alirocumab	Semaglutide
Dosing_Frequency	Twice yearly	Biweekly/Monthly	Biweekly	Weekly
Administration_Mode	Clinician injection	Self-injection	Self-injection	Self-injection
Setting	RCT	RCT	RCT	RCT
Study_Design	Pooled Phase 3	FOURIER	ODYSSEY OUTCOMES	STEP 1
Sample_Size	3454	27,564	18,924	1961
Follow_Up	18–36 months	2.2 years	2.8 years	68 weeks
Adherence_Measured	Yes	Yes	Yes	Yes
Adherence_Metric	Scheduled injections	Persistence (trial)	Trial adherence	Trial adherence
Adherence_Result	High (trial controlled)	High in trial	High	High
Lifestyle_Measured	No	No	No	Yes
Lifestyle_Details	Not reported	Not reported	Not reported	Diet + exercise
Behavioral_Substitution_Assessed	No	No	No	No
Primary_Outcome	LDL-C reduction	CV events	CV events	Weight loss
Notes	Lifestyle not assessed	No lifestyle metrics	Lifestyle not measured	Lifestyle intervention

**Table 2:** Risk of Bias Assessment (RoB 2).

Study	Rand.	Dev.	Miss.	Meas.	Rep.
Ray 2023 [4]	Low	Low	Low	Low	Low
Sabatine 2017 [1]	Low	Low	Low	Low	Low
Schwartz 2018 [2]	Low	Low	Low	Low	Low
Wilding 2021 [5]	Low	Some concerns	Low	Low	Low

Rand. = Randomization; Dev. = Deviations from intended interventions; Miss. = Missing outcome data; Meas. = Measurement of outcomes; Rep. = Selection of reported results.

**Table 3:** Lifestyle Measurement by Therapy Class.

Therapy Class	Diet Measured (%)	Physical Activity Measured (%)	Structured Behavioral Intervention (%)
GLP-1 RA	100	100	100
PCSK9 mAb	0	0	0
PCSK9 siRNA	0	0	0

## Data Availability

The datasets used and/or analyzed during the current study are available from the corresponding author on reasonable request.

## Appendix A. Search Strategy

**PubMed:** (“PCSK9 inhibitors” OR evolocumab OR alirocumab OR inclisiran OR “GLP-1 receptor agonist” OR semaglutide OR liraglutide) AND (“medication adherence” OR persistence OR “treatment adherence”) AND (diet OR “physical activity” OR lifestyle OR “behavioral change” OR “risk compensation” OR “moral hazard”)

**Embase:** (‘PCSK9 inhibitor*’:ti,ab OR evolocumab:ti,ab OR alirocumab:ti,ab OR inclisiran:ti,ab OR ‘GLP-1 receptor agonist*’:ti,ab OR semaglutide:ti,ab OR liraglutide:ti,ab) AND (‘medication adherence’:ti,ab OR adherence:ti,ab OR persistence:ti,ab OR ‘treatment adherence’:ti,ab) AND (diet:ti,ab OR ‘physical activity’:ti,ab OR lifestyle:ti,ab OR ‘behavior change’:ti,ab OR ‘behavioral change’:ti,ab OR ‘risk compensation’:ti,ab OR ‘moral hazard’:ti,ab)

**Web of Science:** TS = ((“PCSK9 inhibitor*” OR evolocumab OR alirocumab OR inclisiran OR “GLP-1 receptor agonist*” OR semaglutide OR liraglutide) AND (“medication adherence” OR adherence OR persistence OR “treatment adherence”) AND (diet OR “physical activity” OR lifestyle OR “behavior change” OR “behavioral change” OR “risk compensation” OR “moral hazard”))

**Scopus:** TITLE-ABS-KEY((“PCSK9 inhibitor*” OR evolocumab OR alirocumab OR inclisiran OR “GLP-1 receptor agonist*” OR semaglutide OR liraglutide) AND (“medication adherence” OR adherence OR persistence OR “treatment adherence”) AND (diet OR “physical activity” OR lifestyle OR “behavior change” OR “behavioral change” OR “risk compensation” OR “moral hazard”))
